# Ammonia as a Power

**Published:** 1891-10

**Authors:** 


### AMMONIA AS A POWER.

A most successful test was made recently of the use of ammonia as a motive power to displace steam. The test was the first that has ever been made on a marine engine, and the trial was most satisfactory. An ammonia plant has been fitted out on the tug E. W. Hartley, which made a trip up and down the Schuylkill river, subjecting the new scheme to a practical test. It is known as the Campbell ammonia engine system, and its workings are novel and interesting, not only to the mechanical and scientific circles, but also the laymen of the industrial world.

Any ordinary engine can be converted into a Campbell ammonia engine simply by the addition of a "generator," which is much like a boiler. Steam is used simply for the purpose of heating the aqua ammonia in the generator. The heated ammonia expels a gas, leaving a weak solution of ammonia in the bottom of this boiler-like affair. When, by raising the temperature of the ammonia, sufficient power is generated, the throttle valve is opened, and the gas passes into the cylinder of the engine, and propels the piston rod in every way the same as steam. It is here exhausted the same as steam, but at this point the gas is cooled and conducted back to the generator. Before it reaches the latter vessel it is carried by a "spray coil" to a point where the gas comes in contact with the ammonia solution, which has been rejected from the generator, and here the solution is recharged by absorption and by the natural affinity existing between water and ammonia.

By this means the same body of ammonia is used constantly, exhausting itself only to be recharged with new life and to be returned to the generator. The same is true of the water used. The steam in the generator imparts its heat to the ammonia, and is thereby condensed to be carried back to the boiler to be used again. In the Campbell ammonia engine there is absolutely no waste. On the other hand there is a saving of coal, as the engine can be operated on one-half the amount of fuel. On the Hartley only one of the two furnaces was used, and there was all the speed and pressure that could be desired.

Many advantages are claimed for the Campbell engine. The principal one is that the life of a boiler is more than doubled. The average term of service of a boiler is less than ten years, and a boat is laid up about one-tenth of the time undergoing repairs to the boiler. Because of the uniform purity of the water this is done away with in the Campbell process, and the boat is in constant service. This and the saving of coal are the chief advantages claimed, but not an unimportant one is the dispensing with the many disadvantages of the lubricating oils. These are wholly unnecessary, as the ammonia itself serves as a lubricant.

